# Environmental and genetic modulation of the phenotypic expression of antibiotic resistance

**DOI:** 10.1093/femsre/fux004

**Published:** 2017-03-08

**Authors:** Diarmaid Hughes, Dan I Andersson

**Affiliations:** Department of Medical Biochemistry and Microbiology, Biomedical Center (Box 582), Uppsala University, S-751 23 Uppsala, Sweden

**Keywords:** persisters, pan genome, epistasis, successful clones, heteroresistance, virulence

## Abstract

Antibiotic resistance can be acquired by mutation or horizontal transfer of a resistance gene, and generally an acquired mechanism results in a predictable increase in phenotypic resistance. However, recent findings suggest that the environment and/or the genetic context can modify the phenotypic expression of specific resistance genes/mutations. An important implication from these findings is that a given genotype does not always result in the expected phenotype. This dissociation of genotype and phenotype has important consequences for clinical bacteriology and for our ability to predict resistance phenotypes from genetics and DNA sequences. A related problem concerns the degree to which the genes/mutations currently identified *in vitro* can fully explain the *in vivo* resistance phenotype, or whether there is a significant additional amount of presently unknown mutations/genes (genetic ‘dark matter’) that could contribute to resistance in clinical isolates. Finally, a very important question is whether/how we can identify the genetic features that contribute to making a successful pathogen, and predict why some resistant clones are very successful and spread globally? In this review, we describe different environmental and genetic factors that influence phenotypic expression of antibiotic resistance genes/mutations and how this information is needed to understand why particular resistant clones spread worldwide and to what extent we can use DNA sequences to predict evolutionary success.

## Teaser

The ability to predict resistance phenotypes from DNA sequence data, and to understand the success of problematic resistant clones, depends on having a good understanding of how environmental factors and genetic context modulate the expression of antibiotic resistance phenotypes and clonal success.

## INTRODUCTION AND SCOPE

Our understanding of the mechanisms of antibiotic resistance has increased tremendously in recent decades, and today we have a relatively good understanding of how bacteria evolve both low- and high-level resistance. Resistance mechanisms can be classified into three, sometimes partly overlapping, main categories: (i) *intrinsic resistance* that comprises mechanisms intrinsic to the bacterium, which constrain the action of the drug, for example, slow uptake or extrusion of the antibiotic by efflux pumps; (ii) *acquired resistance* where a mutation or horizontal transfer of a resistance gene confer resistance, typically by modifying/degrading the antibiotic or modifying/protecting the drug target; and (iii) *adaptive resistance*, which we here define as a transient increase in resistance due to induction of a gene by the antibiotic itself, i.e. the interaction with the antibiotic is the trigger of resistance to that antibiotic and sometimes other antibiotics.

In the majority of cases an acquired mechanism, whether by mutation or a resistance gene, typically results in a predictable increase in phenotypic resistance irrespective of bacterial growth conditions or the genetic context. For example, in *Escherichia coli* (and other bacterial species) specific point mutations in the *rpsL, rpoB* or *gyrA* genes always result in an increased level of resistance to streptomycin, rifampicin or nalidixic acid, respectively, independent of growth conditions or genetic background. However, it is becoming increasingly apparent that a specific resistance genotype can be partly/fully disconnected from the phenotype such that the presence of a resistance mutation/gene does not always result in phenotypic resistance. A disassociation of genotype and phenotype could occur at at least two levels: (i) for a given genotype the resistance phenotype might be altered by environmental changes that modify the level of resistance and (ii) for a given resistance mutation/gene the resulting phenotype might be influenced by the genetic context of the resistance determinant.

Another issue with acquired resistance is the degree to which we can hope to fully understand resistance phenotypes in clinical isolates. Our understanding of the relevant genetic information is typically acquired by *in vitro* selection of mutations and the identification of horizontally transferred resistance genes, and then interpreted by making DNA sequence comparisons to the genotypes of resistant clinical isolates. Although we can often show that at least some of the mutations and genes identified *in vitro* are present in resistant clinical isolates, it is much more difficult to determine whether these are the only, or indeed the most relevant, genetic factors involved in the resistance of these strains. In many cases, multiple genetic changes (both mutations and acquired genes), with individually small effects, could make a significant contribution to the clinical resistance phenotype. Also, the phenotype of a resistant clone could be strongly influenced by epistatis and interactions between resistances, or by genetically unstable resistances.

A related issue is the degree to which we can ascribe the success and prevalence of a resistant clinical isolate to its resistance genetics *per se* rather to other genetic features that contribute to making it a successful pathogen. There is now overwhelming evidence that some combinations of genetic features in particular bacterial lineages, sometimes independent of their resistance genetics, make a very important contribution to the success of several globally problematic antibiotic resistant strains.

In this review, we will describe several such examples of genotype–phenotype disassociations, their mechanisms and consequences for clinical bacteriology. The importance of understanding the responsible mechanisms is relevant for comparisons of resistance phenotypes *in vitro* and *in vivo* and for understanding why particular clones spread worldwide. For example, will a resistant phenotype *in vitro* necessarily cause resistance *in vivo* and conversely will a susceptible phenotype in the laboratory also be susceptible during growth in a host. To what degree does the *in vitro*-determined genetics of strong effect mutations (and genes) explain resistance or is there also a significant additional ’dark matter’ of genetics contributing to *in vivo* phenotypes. Can we understand, and predict, why some resistant clones are very successful and spread globally? Answers to these questions are central to determining and evaluating the efficacy and use of antibiotics in clinical settings based on *in vitro* and animal tests as well as to evaluate the feasibility of genotype-based methods (i.e. PCR and DNA sequencing) to replace phenotypic methods for susceptibility testing of patient samples in clinical environments.

## ENVIRONMENTAL MODULATION OF RESISTANCE

One obvious conclusion from a century of genetic and physiological studies is that environmental conditions can drastically modify and change the phenotypic expression of a specific genotype/gene. That this is also the case for mutations/genes that confer antibiotic resistance has become clear during the last few decades and even though many resistances show full penetrance, and are largely independent of environmental conditions, others might exhibit a strong dependence on environmental and growth conditions. Below we discuss five potential ways (A–E) in which the environment could modulate the phenotypic effect of a resistance mutation/gene. This division is somewhat arbitrary and certain pathways can be placed under several sections depending on your perspective.

### Collective resistance

**Figure 1.**
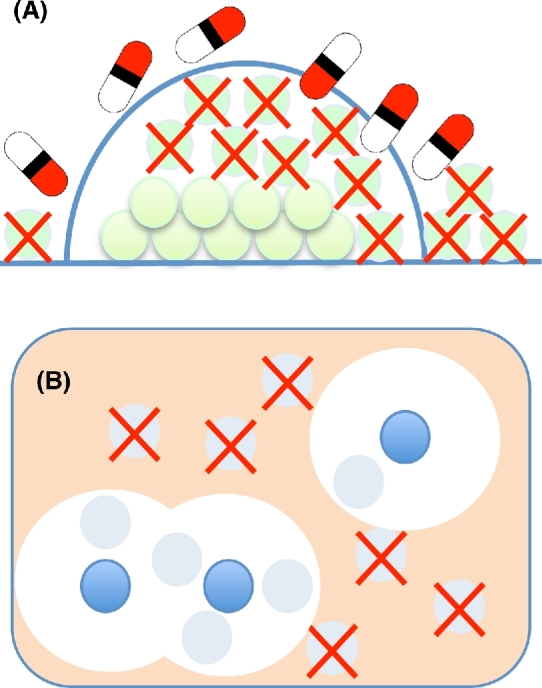
Collective resistance to antibiotics. (**A)** Bacteria in biofilms have greater resistance to antibiotics (due to the combined effects of physical protection and altered growth physiology) than planktonic bacteria. Dead bacteria are shown with an crosses; live bacteria are shown as green circles. (**B)** Indirect resistance can occur when some bacteria in an environment reduce the active antibiotic concentration. Antibiotic is shown as light brown colour, bacteria causing antibiotic inactivation shown as blue, zone of antibiotic inactivation shown as white circles. Antibiotic inactivation provides protection to susceptible bacteria in the antibiotic-free environment (grey bacteria within the white zones). Dead bacteria are indicated by grey circles with a red X.

These are mechanisms where the phenotypic behaviour of a bacterium is modified due to formation of communities of bacteria of different or the same species, e.g. biofilm formation and indirect resistance (Fig. 1).

#### Biofilms

Antibiotic resistance in bacterial biofilms has been covered in several recent reviews (Hoiby *et al.*^2010^; Jolivet-Gougeon and Bonnaure-Mallet 2014; Taylor, Yeung and Hancock 2014; Olsen 2015), and we will only briefly summarise the mechanisms involved. Bacterial biofilms consist of bacteria (same or different species) that are attached to foreign bodies or natural surfaces where the cells are encased in a self-produced matrix of extracellular compounds, including polysaccharides, proteins and DNA. Compared to the planktonic state bacteria might show a 100- to 1000-fold increase in resistance to antibiotics (Ceri *et al.*^1999^) and underlying this resistance is a number of different mechanisms that operate together to confer high-level resistance. These include, in comparison to planktonic cells, altered metabolism, high cell density, spatial structure and presence of extracellular compounds. It should also be noted that cells within a biofilm show physiological heterogeneity and that different resistance mechanisms might operate for different cells in the biofilm. One mechanism involves the inability of antibiotics to penetrate the biofilm (Fig. 1A) and this affects positively charged peptides and aminoglycosides that can be sequestered in the negatively charged extracellular matrix (Walters *et al.*^2003^; Chiang *et al.*^2013^). A more significant mechanism involves various starvation conditions induced in the biofilm due to spatial heterogeneity in levels of, for example, nutrients and oxygen that cause slow growth and are well known to generally increase antibiotic tolerance. Thus, increased tolerance is expected for cells with low metabolic activity and slow growth located in the inner parts of the biofilm (Bernier *et al.*^2013^; Ciofu *et al.*^2015^). Furthermore, oxygen deprivation deep in biofilms is extensive and hypoxia might cause increased resistance by altered efflux regulation (Borriello *et al.*^2004^; Schaible, Taylor and Schaffer 2012) or downregulation of energy metabolism and reduced drug uptake (Allison, Brynildsen and Collins 2011; Taylor, Yeung and Hancock 2014). A third broad mechanism involves the induction of general stress responses and/or specific genes that confer resistance. For example, induction of RpoS-dependent (Ito *et al.*^2009^), LexA-dependent responses (Bernier *et al.*^2013^) and quorum-sensing responses (Shih and Huang 2002; Bjarnsholt *et al.*^2005^) in biofilms can increase resistance to ampicillin, fluoroquinolones and aminoglycosides, respectively. These general responses together with biofilm-specific induction of genes involved in, for example, glucan biosynthesis (Mah *et al.*^2003^), type VI secretion (Zhang *et al.*^2011^), efflux pumps (Gillis *et al.*^2005^) and gene regulation (Lynch *et al.*^2007^; Liao and Sauer 2012; Liao, Schurr and Sauer 2013) are important contributors to resistance. In most cases, the specific inducers of these responses are unknown but it is conceivable that increased levels of various metabolites produced by starved cells in the biofilm are involved (Helling *et al.*^2002^).

#### Indirect resistance

Indirect resistance (IR), also known as passive resistance and cooperative resistance, is the ability of a population of antibiotic-resistant bacteria to protect a population of susceptible bacteria (Fig. 1B). Clinically, the relevance of IR is when an antibiotic-resistant population can protect a pathogenic antibiotic-susceptible population that is the intended target of the antimicrobial treatment (Maddocks and May 1969; Brook 2009). The majority of described cases of IR involves bacteria-producing β-lactamases where the enzyme is located in the periplasm or excreted in the medium where it remains active (Georgiou, Shuler and Wilson 1988). The periplasmic and excreted β-lactamases act by reducing the antibiotic concentration in the surrounding environment, thereby allowing susceptible cells nearby to survive. Apart from β-lactamases, recent work has shown that other types of antibiotic modifying/degrading enzymes can also confer IR (Nicoloff and Andersson 2016). Thus, bacteria expressing the antibiotic-modifying or antibiotic-degrading enzymes Ere(A), Tet(X2) or CatA1 caused IR in presence of macrolides, tetracyclines and chloramphenicol, respectively, indicating that IR has a broader relevance and that antibiotic resistant co-infecting bacteria or the normal microflora might exert a considerable negative effect on the efficacy of antimicrobial therapy.

IR can also occur when susceptible cells, instead of expressing a degradation or modification enzyme, by themselves might act as a physical ‘sink’ to sequester and reduce the free concentration of drug. In such cases, the efficacy of antibiotic treatment depends on the amount of antibiotic per bacterial cell and this level will determine whether growth is inhibited or not. This type of IR has been observed for aminoglycosides (Tan *et al.*^2012^) but could conceivably be relevant for any antibiotic that binds strongly to a cellular organelle (e.g. ribosome) or other component.

IR might be thought of a common goods problem where the susceptible bacteria are regarded as ‘cheaters’, provided the resistance mechanism confers a cost (Yurtsev *et al.*^2013^). In such a situation, the dynamics of the mixed population will depend on the cost of the resistance, the antibiotic concentration and the minimal selective concentrations (MSC) of the susceptible and resistant bacteria. Thus, at high concentrations (above MSC), the resistant bacteria grow faster and will take over whereas below the MSC the cost of the resistance mechanism results in enrichment of the susceptible bacteria.

### Changes in resistance due to altered growth states and physiology

**Figure 2.**
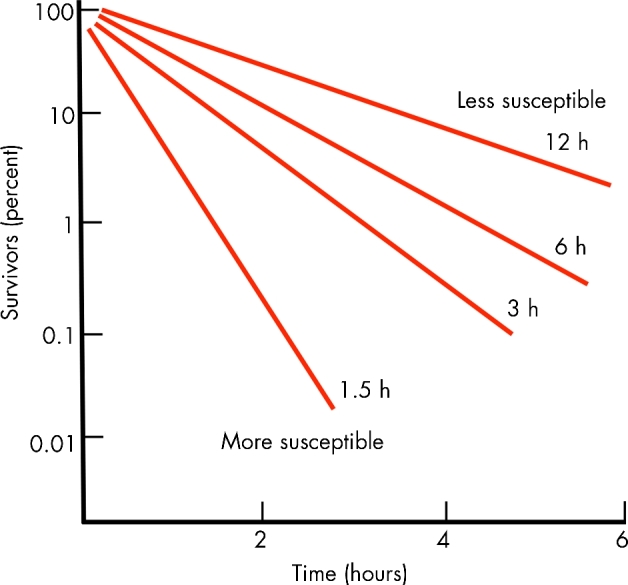
Antibiotic resistance is influenced by growth physiology. Bacteria growing faster are usually more susceptible to antibiotic inhibition than bacteria that are slow growing or in stationary phase. The figure illustrates relative susceptibility of *E. coli* during chemostat growth to killing by a cephalosporin as a function of bacterial generation time (1.5–12 h). Slower growing cultures are less susceptible to the antibiotic than faster growing cultures. The data are adapted from Tuomanen *et al*. (1986). As described in the text, many different physiological and environmental factors associated with slow growth/stationary phase could, individually or in combination, make bacteria more refractory to antibiotics.

These are cases where an alteration in growth rate or the presence of a specific metabolite/compound modifies resistance (Fig. 2).

#### Factors affecting growth rate

It has been known for a long time that the growth rate of the bacteria has a strong influence on antibiotic susceptibility and already in 1944 (Lee, Foley and Epstein 1944) it was shown that slow growth reduced the efficacy of penicillin and other β-lactam antibiotics (Tuomanen *et al.*^1986^). This observation was the foundation for the subsequent and much used penicillin enrichment method to select for conditionally lethal mutants (Lederberg and Zinder 1948). Similar effects on antibiotic susceptibility by growth rate/metabolic state have been shown for several other antibiotic classes, including tetracyclines (Levin and Rozen 2006), isoniazide (Koul *et al.*^2011^), metronidazole (Ginsberg 2010) and other drugs (Pethe *et al.*^2010^). However, other drug classes seem to be largely indifferent to the growth state with regard to their inhibitory/killing capacity (e.g. fluoroquinolones and aminoglycosides), even though also for these drugs killing is less pronounced for stationary phase cells compared to exponentially growing (Stenstrom, Conway and Kjelleberg 1989; Levin and Rozen 2006). Since alterations in growth rate will have many different and complementary effects on bacterial physiology, it has been difficult to pin point the specific underlying mechanisms for the increased resistance of slow/non-growing cells. However, one signal that seems to be relevant for increased resistance in several bacterial species is the alarmone (p)ppGpp, which is the effector molecule of the stringent response and growth rate regulation in bacteria (Srivatsan and Wang 2008) The accumulation of (p)ppGpp is involved in downregulating the protein-synthetic machinery and upregulating biosynthetic capability, and it also triggers responses to increase antibiotic tolerance (Abranches *et al.*^2009^; Wu, Long and Xie 2010). Other key regulators (whose activity could be modulated by growth rate) involved in antibiotic resistance are, for example, H-NS in *E. coli* (Nishino, Hayashi-Nishino and Yamaguchi 2009), two-component regulators such as PhoPQ in *Enterobacteriaceae* (Hirakawa *et al.*^2003^; Gooderham and Hancock 2009), CbrAB in *Pseudomonas aeruginosa* (Yeung, Bains and Hancock 2011) and EnvZ-OmpR in Gram negatives (Mortimer and Piddock 1993; Adler *et al.*^2012^).

#### Causes of increased resistance

A key question is what is the ultimate cause of increased resistance in slow-growing cells. In some cases, e.g. in the PhoPQ system, the ultimate causation is an alteration in lipid A that reduces negative charge and which results in electrostatic repulsion of cationic peptides to the cell wall (Bauer and Shafer 2015). Another common response, similar to what is observed in biofilms, is the upregulation of multidrug-resistant (MDR) efflux pumps that increase extrusion of antibiotics (Li, Plesiat and Nikaido 2015) or reduced drug uptake (associated with reduced metabolic activity and/or alterations in porine composition) (Delcour 2009; Mckay and Portnoy 2015). Furthermore, upregulation of specific chromosomally encoded resistance genes like *ampC* might also increase resistance in growth-dependent manner (Jacoby 2009). However, in a majority cases we do not know in detail how an altered growth rate/metabolic state confers increased resistance. Other less explored mechanisms include growth rate/metabolic state-dependent target modifications (e.g. methylations of rRNA and ribosomal proteins), expression of promiscuous enzymes having trace activities of antibiotic modification/degradation or upregulation of bona fide ‘cryptic’ resistance genes. The latter has been described in *Salmonella* Typhimurium LT2 where the chromosomally located *aadA* gene, encoding an aminoglycoside modifying enzyme conferring streptomycin resistance, is turned off in rich media. However, under certain conditions where electron transport is impaired or the growth rate is reduced (e.g. growth on glycerol as carbon source), this gene is turned on resulting in a 30-fold increase in minimal inhibitory concentration (MIC) of streptomycin. Expression of the *aadA* gene is positively regulated by the stringent response regulator (p)ppGpp and these results show how slow-growth environments (or mutations that confer slow growth by impairing electron transport) that increase (p)ppGpp levels can activate a gene that is usually silent (Koskiniemi *et al.*^2011^).

#### Possible role of oxygen

Another potentially relevant mechanism of modulation of antibiotic killing involves levels of oxygen, increased respiration and formation of oxygen radicals. Studies from Collins group (Kohanski *et al.*^2007^; Dwyer *et al.*^2014^; Lobritz *et al.*^2015^) suggested that killing of cells by the bactericidal antibiotic classes fluoroquinolones, aminoglycosides and beta-lactams is mediated via a common pathway of production of toxic hydroxyl radicals. Thus, these authors suggested that these antibiotic classes all cause a depletion of NADH which results in hyperactivation of the electron transport chain which in turn stimulates production of superoxide ions. These ions damage Fe-S clusters, making ferrous ions available for oxidation via the Fenton reaction and subsequent formation of hydroxyl radicals that damage various cellular components and causes death. If correct, this idea suggests that oxygen levels could be a significant modulator of antibiotic susceptibility. However, this hypothesis has been strongly challenged by other researchers who suggest that several predictions of this model are not fulfilled, e.g. there is no increase in superoxide or free iron levels by antibiotic treatment, antibiotic lethality persists in absence of oxygen and DNA repair mutants do not show hypersensitivity to antibiotics (Keren *et al.*^2013^; Liu and Imlay 2013). In spite of these objections, a recent study showed that high oxygen levels can increase antibiotic susceptibility but whether this effect involves the killing process described above remains unclear (Kolpen *et al.*^2016^).

#### Host factors

A recent interesting study demonstrated how host-specific growth conditions might cause a transient increase in antibiotic resistance to polymyxin B (>100-fold) that is mediated by PhoPQ/PmrAB system. These authors showed for S. Typhimurium that during growth *in vitro* in medium mimicking conditions within a phagosome (low pH, phosphate and magnesium) and during growth in macrophages and certain tissues of mice, the bacteria were significantly more resistant than during growth in standard laboratory media (Kubicek-Sutherland *et al.*^2015^). Considering the known regulatory role of the PhoPQ/PmrAB in resistance to antimicrobial peptides, it is likely that the resistance is conferred via the ArnBCADTEF and PmrCE proteins that are involved in modifying lipid A by additions of 4-amino-4-deoxy-l-arabinose and phosphoethanolamine that cause a reduction in the net negative charge of the LPS and electrostatic repulsion of cationic peptides (Matamouros and Miller 2015). The most important implication from this study is that growth inside a host can drastically alter resistance levels and thus, standard *in vitro* testing of antibiotic susceptibility (MIC levels) might not correctly reflect the resistance level of the pathogen inside the relevant host and tissue.

### Antibiotic-induced resistances

**Figure 3.**
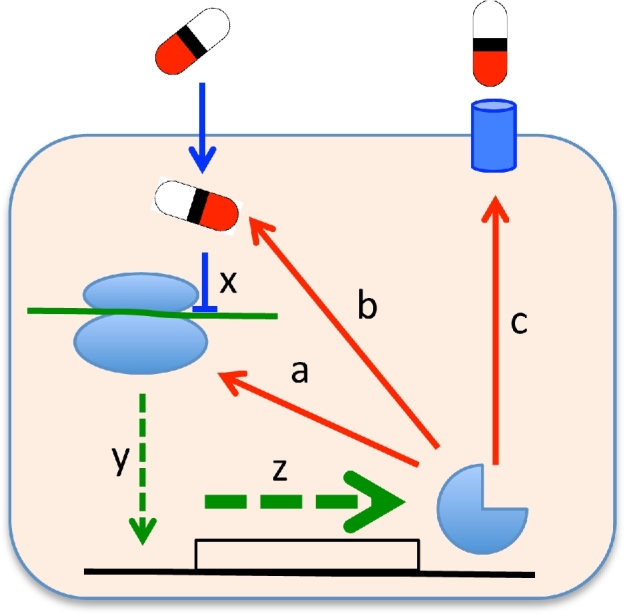
Antibiotic- or metabolite-induced resistance. Inhibition of translation or transcription (x) by an antibiotic (e.g. a macrolide) can cause production of proteins/enzymes (y, z), which increase resistance to the antibiotic by one of several mechanisms: (**a**) modification of the polymerase to make it resistant to the antibiotic, (**b**) inactivation of the antibiotic, (**c**) increased expression of an efflux pump to reduce the antibiotic concentration. Metabolites (environmental or produced as a consequence of antibiotic-associated disruption of normal metabolism) could also increase resistance by various mechanisms, including by causing increased expression of efflux pumps.

This type of mechanism can be classified as adaptive resistance where antibiotics cause induction of various types of resistance mechanisms that confer resistance to the specific inducer and sometimes also other antibiotics (Fig. 3). This type of resistance mechanism can sometimes be confused with other mechanisms. Thus, enrichment of resistant bacteria in response to a specific antibiotic could occur by at least four different mechanisms: (i) induction of specific resistance pathway (the focus of this section), (ii) enrichment of low-frequency phenotypic variants where the resistance has a non-genetic basis, (iii) enrichment of low-frequency highly unstable genetic variants where the basis for the resistance is genetic but the mutation type is frequent in a population and highly unstable and (iv) enrichment of pre-existing common genetically stable resistant mutants. The typical test for adaptive resistance involves growth of an initially susceptible population of bacteria at non-lethal antibiotic concentrations and demonstration that the surviving and enriched bacteria are resistant. Subsequent growth in absence of antibiotic should result in rapid loss of resistance. If the latter is not observed, mechanism (iv) is likely to be involved since the resistant cells appear genetically stable. The frequency of the resistant population after initial exposure is expected to be high (100%) for mechanism (i), whereas for (ii) and (iii) it is small subpopulations that show the resistance phenotype and which are enriched after antibiotic exposure. This provides a criterion to distinguish (i) from (ii) and (iii). However, mechanisms (ii) and (iii) are not easily distinguishable since certain mutation types (gene amplification) have dynamic properties—high frequency and high instability (reversibility)—that are very similar to true non-genetic phenotypic variability. These two mechanisms will be discussed further below under the section ‘Stochastic switches’.

Antibiotic-induced resistance has been observed for several antibiotic classes in both Gram negatives and positives. In Gram positives, antibiotics that inhibit ribosome function, including tetracycline, chloramphenicol and macrolides, confer induced resistance by virtue of target modification or by drug efflux (see Depardieu *et al.*^2007^; Chancey, Zahner and Stephens 2012 for overviews). A well-defined mechanism for macrolides involves induction of methylases encoded by the *erm(A), erm(B)* and *erm(C)* genes that modify one specific adenine in the 23S rRNA, resulting in blockage of antibiotic binding. These genes are widespread among Gram-positive genera and can be found in, for example, Staphylococci, Enterococci and Streptococci (Roberts 2008). All these three genes are regulated by translational attenuation where binding of the drug to the exit tunnel of the ribosome results in leader peptide-mediated stalling of the ribosome (Horinouchi and Weisblum 1980; Mayford and Weisblum 1989; Vazquez-Laslop, Thum and Mankin 2008; Ramu, Mankin and Vazquez-Laslop 2009). In this mechanism of translational attenuation, the stalling results in formation of an mRNA secondary structure of the *erm* gene that precludes the sequestering of the ribosomal binding site (RBS), which normally occurs in the absence of drug. When the RBS is present in single-stranded form, ribosomes can access and activate *erm* gene translation. Whether the transcript is in inactive or active translation form thus depends on the absence or presence of the antibiotic. The *erm* genes show variation in the details of the attenuation site, the leader peptide sequence and length and in the specificity of different macrolides to cause induction but the basic mechanism of attenuation appears the same. In addition to translational attenuation, the *erm(K)* gene in *Bacillus* is regulated by a macrolide-dependent transcriptional attenuation where alternative mRNA structures either leads to transcriptional readthrough (no termination structures formed) in the presence of drug or to transcriptional termination (termination structures formed) in the absence of drug (Kwon *et al.*^2006^). Similar cases of transcriptional attenuation also exist for induction of efflux systems in various Gram positives (Ojo *et al.*^2006^; Chancey *et al.*^2011^; Le Bouter, Leclercq and Cattoir 2011). A recent paper used a new screening method to search for riboswitches/regulators in *Bacillus subtilis* and *Listeria monocytogenes*. Several known but also new antibiotic-responsive riboregulators were discovered, indicating that termination-based regulation of antibiotic resistance genes is common in Gram-positive bacteria and that several new systems are awaiting discovery (Dar *et al.*^2016^).

Other well-described examples of inducible resistance involve vancomycin resistance in Gram positives (Depardieu *et al.*^2007^; Perichon and Courvalin 2009) and *ampC*-dependent ampicillin resistance in Gram negatives (Hanson and Sanders 1999; Jacoby 2009). These systems have been described extensively in recent papers and will not be further discussed here. One key question in this context is why certain resistances are inducible? A likely explanation for this is the fitness costs associated with expression. Thus, it has been shown that there is a considerable cost of expressing methylases (Lioy *et al.*^2014^), the *vanA* operon (Foucault *et al.*^2010^) and the *ampC* gene (Morosini *et al.*^2000^), and it is expected that such a cost will provide a strong selection pressure to evolve an inducible mechanism.

### Metabolite-modulated resistances

These types of mechanisms have no common underlying mechanism but they are examples where a specific metabolite (other than an antibiotic) can modulate the resistance phenotype. An interesting case is the fully reversible resistance phenotype of the clinically relevant *cysB* mutants that are highly mecillinam (named Amdinocillin in the USA) resistant. The MIC of mecillinam is increased from 0.125 to 24 ug/ml by the *cysB* mutation. Mecillinam is a β-lactam antibiotic that inhibits PBP2 function and which is exclusively used for uncomplicated urinary tract infections (Nicolle 2000). By adding cysteine to the growth medium, the resistant phenotype is completely reversed and the MIC of mecillinam drops to wild-type level (Thulin, Sundqvist and Andersson 2015). The underlying mechanism for this is unknown but an interesting implication is that resistance may be abrogated by certain metabolites. Two other interesting examples of metabolite effects on resistance phenotypes are those of metabolite-enabled eradication of persisters by aminoglycosides (Allison, Brynildsen and Collins 2011) and nitrogen oxide-induced resistance to aminoglycosides (McCollister *et al.*^2011^). In the former case, it is proposed that presence of certain sugars can increase proton motive force and in doing so potentiate the effect of aminoglycosides by increasing drug uptake. In the latter case, nitric oxide appears to block respiration and the energy-dependent phases of aminoglycoside uptake, and thereby reduce drug susceptibility. This implies that host inflammatory responses associated with infection can promote resistance to aminoglycosides. Also, specific metabolites might have strong effects on drug efflux pumps and cause their upregulation. For example, intracellular metabolites as well as extracellular compounds that are not antibiotics can cause induction of pumps and make the cells more resistant. For example, metabolites in amino acid biosynthetic pathways that might accumulate under certain conditions (Helling *et al.*^2002^) or external compounds and pharmaceuticals that the bacteria might be exposed to in a treated patient (Cohen *et al.*^1993^) could potentially increase resistance levels.

### Persisters and stochastic switches

With regard to phenotypic and growth-state-dependent tolerance to antibiotics, there exists a whole spectrum of different phenomena (stationary phase, biofilms, dormancy, persisters, etc.) that based on their different genetic characteristics and dynamics are likely to be different processes even though the phenotypic outcome is similar in generating antibiotic tolerance (see a good review in Kester and Fortune 2014). Persisters represent a special case and here they are defined as a rare phenotypic variant that survives extended exposure to antibiotic concentrations above the MIC of the antibiotic. Thus, after the addition of antibiotic to a culture, the majority of cells dies quickly but a small fraction (typically 10^−6^ to 10^−3^) survives. After the restart of growth, this surviving population of persisters behaves as the original population. From the early work of Moyed and Bertrand (1983) and later several other groups, it has become clear that toxin–antitoxin systems play a major role as effectors of the persistent state (reviewed in Page and Peti 2016). However, other mechanisms are probably also involved as shown by the role of the *glpD* gene (encodes an enzyme involved in glucose utilisation) in metabolically induced persistence (Girgis, Harris and Tavazoie 2012), and it has been suggested that persistent cells might be generated by a variety of mechanisms and pathways whose common characteristic is that they stop growth in a reversible manner. As discussed by Levin, Concepcion-Acevedo and Udekwu (2014), one view of persistence is: ‘not an evolved (selected-for) character but rather like mutation, an inadvertent product of different kinds of errors and glitches’. With this view, the various genetic systems identified (i.e. toxin–antitoxin systems) are modifiers of the rate by which these errors occur.

Two key questions that have been less addressed are the clinical relevance of persisters and which mechanisms, in a supposedly genetically homogenous population, are involved in generating variation in levels of, for example, a toxin–antitoxin system that causes growth arrest. With regard to the former question, it is clear that, for example, biofilms and dormancy in *Mycobacterium tuberculosis* are involved in chronic and recurrent infections, but it still remains unclear if a small fraction of planktonic persisters (as defined here) plays any role in clinical settings. One argument that they might be less important than often argued is the fact that bacteriostatic antibiotics do in fact work at all, and generally as efficiently as bacteriocidal drugs (Nemeth, Oesch and Kuster 2015). A bacteriostatic antibiotic is basically a drug that converts 100% of the population into a non-growing (persistent) state, and with this kind of reasoning it is difficult to see why a small fraction of persisters would be problematic.

With regard to the underlying mechanisms that generate the heterogeneity allowing a subpopulation to enter a persistent state, it is usually assumed that it is caused by some type of stochastic switch and/or noise in metabolite/protein/RNA level that is non-genetic. This argument is based on the relatively high frequency of persisters (10^−6^ to 10^−3^) in a growing population and the rapid reversibility of the phenotype (after overnight growth persisters have exited the state), which is very different from the lower frequency and non-reversibility of typical mutations. However, an explanation that has (to our knowledge) not been extensively explored is that the underlying mechanism is genetic and caused by a specific class of mutations, i.e. gene duplications/amplifications. Thus, these mutations have characteristics that are very similar to what is observed for persisters. That is, they are very common in a population with frequencies in the range of 10^−5^ to 10^−2^ (Anderson and Roth 1981; Andersson and Hughes 2009). Furthermore, duplications/amplifications are highly unstable (Pettersson *et al.*^2008^; Andersson and Hughes 2009; Reams *et al.*^2010^) and can be lost at rates similar to rate by which persisters exit the non-growing state. Based on this argument, a conceivable mechanism for generation of heterogeneity in a population could be the transient and unstable formation of duplications/amplifications that cause fluctuations in, for example, toxin–antitoxins or any other RNA/protein that could be involved in growth arrest.

## GENETIC CONTEXT AND RESISTANCE PHENOTYPE

After decades of genetic analysis of antibiotic resistance, both *in vitro*-selected strains and clinical isolates, it has become obvious that there is very frequently an interplay between multiple genetic alterations that is involved in shaping a resistance phenotype. This can be because multiple mutations of small affect in different (or acquired HGT genes) contribute via a variety of mechanisms (e.g. target modification versus drug influx/efflux) to the observed reduction in susceptibility, or because additional mutations are selected to reduce the fitness coasts caused by primary resistance mutations, or because features of the genome not directly related to resistance contribute to clonal success (e.g. virulence or transmission). Below we discuss ways in which the genotype complexity could modulate the expression of a resistance phenotype, and the success of resistant clones.

### Resistance usually requires multiple genetic alterations

**Figure 4.**
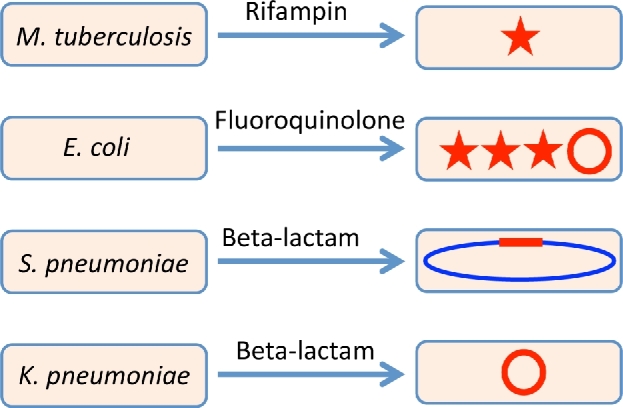
Genetic resistance mechanisms as a function of drug and bug. The relative importance of different resistance mechanisms depends on both the bacterial species and the drug class. Mutations are indicated by a red star (one mutations is sufficient to confer resistance to rifampicin in *M. tuberculosis*, whereas multiple mutations are required to confer resistance to fluoroquinolones in *E. coli*). Plasmid-borne resistance (indicated by a red circle) can also contribute to fluoroquinolone resistance, and is the major mechanism of resistance to β-lactam antibiotics in most species, including *K. pneumoniae*. In contrast, in some species (Streptococcal spp., and Neisseria spp.), the creation of mosaic PBP genes by HGT (indicated by a red section on the chromosome) is the major mechanism of resistance to β-lactam antibiotics.

There are some examples where clinical resistance to an antibiotic can be caused by single chromosomal mutations (Fig. 4). The classic example is resistance to antibiotics used to treat *Mycobacterium tuberculosis* (Mishra *et al.*^2015^). *Mycobacterium tuberculosis* is unusual among the pathogens treated with antibacterial drugs in that it is a relatively new species bacterial species that exclusively infects humans, and its genome is almost completely refractory to HGT (Eldholm and Balloux 2016). These features have resulted in a species that displays very little genetic variability between independent isolates (Cubillos-Ruiz, Morales and Zambrano 2008; Casali *et al.*^2012^; Comas *et al.*^2012^). Resistance to rifampicin, one of the most important first-line antituberculosis drugs, is exclusively associated with mutations in *rpoB*, usually a single mutation per isolate (Casali *et al.*^2012^; Comas *et al.*^2012^; Brandis *et al.*^2015^). A complicating factor is that the resistance mutations in *rpoB* typically have an associated fitness cost. In clinical *M. tuberculosis* isolates, many resistant strains were found by whole genome sequencing (WGS) to carry additional mutations (genetic ‘dark matter’) in genes coding for other subunits of the RNAP, and it was speculated that at least some of these might be fitness-compensatory mutations (Casali *et al.*^2012^; Comas *et al.*^2012^). This hypothesis was supported by experimental evolution and genetic reconstruction experiments, using *Salmonella enterica* as a model organism, where it was shown that many different secondary mutations in either *rpoA*, *rpoB* or *rpoC*, encoding different subunits of RNAP, could compensate the fitness costs of primary resistance mutations in *rpoB* (Brandis *et al.*^2012^; Brandis and Hughes 2013). The genotype–phenotype relationship in *M. tuberculosis* has also been studied for aminoglycosides and the data, based on comparison of *in vitro*-generated and clinical data, suggest that for these drugs there is also a direct relationship between single chromosomal mutations and antibiotic resistance (Prammananan *et al.*^1998^; Shcherbakov *et al.*^2010^). In summary, the genotype–phenotype relationship describing resistance to rifampicin and aminglycosides in *M. tuberculosis* seems to be very direct, with a strong and simple correlation between *in vitro*-generated data and clinical resistance data. The genetic homogeneity of the species, and the lack of contribution by HGT, may together simplify the genotype–phenotype relationship describing antibacterial drug resistance in *M. tuberculosis*. *Mycobacterium tuberculosis* continues to be challenging to study using genetic methods (Shcherbakov *et al.*^2010^; Nebenzahl-Guimaraes *et al.*^2014^), but given the genetic homogeneity of this species, a feasible approach to a fuller understanding of its resistance phenotypes and pathogenic potential would be to carry out WGS of clinical isolates on a massive scale and apply genome-wide association studies to analyse the data in relation to phenotypic characterisation of resistance and epidemiology, to identify the primary and ancillary mutations contributing to resistance and transmissibility (Hatherell *et al.*^2016^; Phelan *et al.*^2016^).

Resistance development to fluoroquinolones (Fig. 4), one of the most frequently used classes of antimicrobials for treating Gram-negative infections (Hooper 2001; Adriaenssens *et al.*^2011^), is much more complex than resistance development in *M. tuberculosis*. It involves multiple mutations, usually affecting both drug target genes and drug efflux regulators, as well as genes acquired by HGT (Webber and Piddock 2001; Komp Lindgren, Karlsson and Hughes 2003; Jacoby 2005). There is apparently no individual genetic change that can increase the MIC of the drug beyond the clinical breakpoint for successful therapy (Marcusson, Frimodt-Moller and Hughes 2009). This raises an important question: How can resistance be selected clinically if it requires multiple independent genetic alterations in order to increase MIC above the clinical breakpoint? Exposing susceptible bacteria to sub-MIC concentrations of antibiotics selects for genetic changes that reduce susceptibility (Gullberg *et al.*^2011^; Hughes and Andersson 2012). The clinical evolution of resistance to fluoroquinolones is consistent with a multistep selection of successively more resistant isolates accumulating multiple genetic alterations (Komp Lindgren, Karlsson and Hughes 2003; Komp Lindgren *et al.*^2005^). In the clinical environment, sub-MIC selection might be caused by unequal drug concentrations in the body during therapy, by the use of suboptimal dosing regimens or of substandard drug preparations, and also by the presence of selective concentrations of drug polluting the wider environment after excretion from patients or after use outside of human medicine (Negri *et al.*^2000^; Andersson and Hughes 2014). We do not know how much each of the above types of exposure has actually contributed to the evolution of fluoroquinolone resistance in clinical isolates. Now that we are aware of the genetic complexity of fluoroquinolone resistance, one can ask whether all of the relevant genetic changes have been identified, or whether there are additional contributing genetic alterations still to be discovered? The short answer is that mutations and genes contributing to fluoroquinolone resistance in clinical or *in vitro*-selected isolates have been identified over several decades (Hooper *et al.*^1987^; Hooper 1999; Wang *et al.*^2001^; Tran and Jacoby 2002; Strahilevitz *et al.*^2009^; Sato *et al.*^2013^; Pietsch *et al.*^2017^), and there is no reason to believe that we have yet reached a complete understanding of the mechanisms and genetic alterations that could contribute to resistance. Indeed, the recent discovery of mutations in *rpoB*, which reduce susceptibility to fluoroquinolones by increasing expression of the MdtK efflux pump (Pietsch *et al.*^2017^), is a good example of experimental evolution uncovering of some of the previously unsuspected genetic ‘dark matter’ of resistance.

Resistance to fluoroquinolones is also closely associated with multidrug resistance. One reason for this is that the genetic alterations often include mutations that upregulate one or more multidrug efflux pumps, a feature that contributes to cross-resistance to other classes of antibiotics (Hornsey *et al.*^2010^). In addition, the fluoroquinolone-resistance genes acquired by HGT are typically carried on MDR plasmids, conferring resistance to unrelated antibiotics (Martinez-Martinez, Pascual and Jacoby 1998; Pitout 2012).

Until recently, some of the major knowledge gaps regarding fluoroquinolone resistance concerned the order in which genetic changes are accumulated, and whether resistance is actually selected in the patient or in the wider environment. Recent work, combining genomic analysis of resistant isolates, with experimental evolution and mathematical modelling, has gone a long way towards resolving these issues (Huseby *et al.*^2017^). The authors identified the order in which resistance mutations were selected in the most common clinical genotype, showed that clinical genotypes could be selected over a range of drug selective pressures (compatible with the possibility to select resistance in different environments) and that selection was strongly influenced by the relative fitness of alternative mutations and factors affecting mutation supply. This study provides strong support for the value of experimental evolution and *in vitro* competition assays as tools to trace evolutionary trajectories, and elucidate the interplay of genetic and environmental influences on resistance evolution (Huseby *et al.*^2017^).

The β-lactams (penicillins, cephalosporins, carbapenems, monobactams) are, in terms of total usage, the most important group of antibiotics. Resistance to β-lactam antibiotics in some species (e.g. *Streptococcus* and *Neisseria*) occurs by homologous recombination (Fig. 4) of DNA acquired by transformation from related species (Tapsall 2009; Hakenbeck *et al.*^2012^). Homologous recombination creates so-called mosaic genes encoding novel penicillin-binding proteins (PBPs) conferring resistance to β-lactams in *Streptococcus pneumoniae* and *Neisseria gonorrhoeae*, and sulfonamide-resistant dihydropterate synthase in *N. meningitidis* (Dowson *et al.*^1993^; Sibold *et al.*^1994^; Maiden 1998; Ito *et al.*^2005^; Chi *et al.*^2007^; Ochiai *et al.*^2008^; Hakenbeck *et al.*^2012^; Sauerbier *et al.*^2012^; Straume, Stamsas and Havarstein 2015). The selection of additional mutations within these novel mosaic genes contributes to further increasing resistance to cephalosporins (Johnson *et al.*^2014^). However, in most clinically interesting pathogens resistance to β-lactams is caused by HGT of plasmids carrying genes encoding any one of a large variety of different β-lactamase enzymes (Fig. 4) that degrade the antibiotic (Bush 2013).

The success of β-lactamases in causing antibiotic resistance illustrates one of the great gaps in current knowledge: namely how to predict the transfer and evolvability of potential resistance genes against novel classes of antibiotics that might be introduced into clinical use. The pool of potential resistance genes in the bacterial ‘pan-genome’ (Tettelin *et al.*^2005^) is vast (D’Costa *et al.*^2006^; Sommer, Dantas and Church 2009; Pawlowski *et al.*^2016^). Next generation sequencing and related omics technologies could potentially identify all gene sequences or enzymatic activities in the environmental biome (human, soil, sea, etc.) that might be capable of conferring resistance to novel antibiotics in human pathogens. However, we still lack the knowledge to predict the probability of transfer into clinically relevant species, and the probability that the transferred genes could evolve to express resistance at a clinically relevant level in the novel genetic environment. These currently unknown genes, and their potential to compromise any novel antibiotics developed, are the most significant ‘dark matter’ of resistance

### Epistasis and interactions between resistance genes and mutations influence phenotype

**Figure 5.**
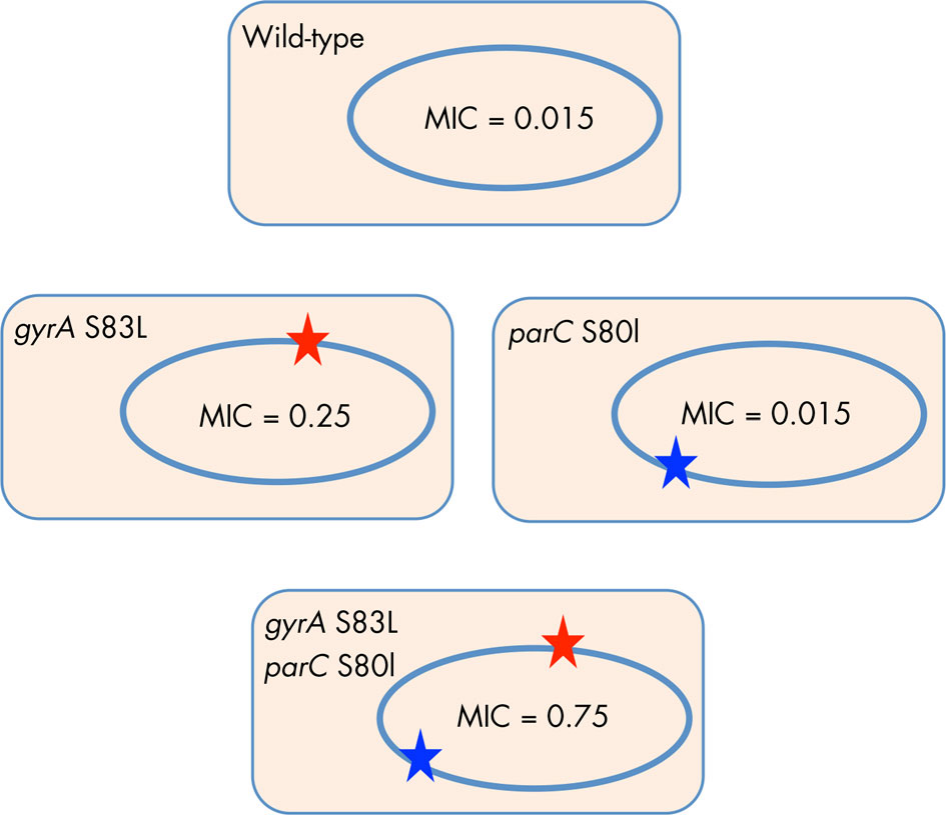
Epistasis can influence antibiotic resistance phenotype. Epistatic interactions between resistance and/or other genes can influence the phenotypic expression of antibiotic resistance (or affect other aspects of bacterial fitness). In this example, *E. coli* MIC for ciprofloxacin (0.015) is increased by a mutation in *gyrA* (red star) but not by a mutation in *parC* (blue star). The double mutant shows clear evidence of epistasis, with an increase in MIC much greater than predicted by additivity. Data from Huseby *et al*. (2017).

Most antibiotic-resistant clinical isolates carry combinations of mutations or acquired genes that together confer resistance to more than one antibiotic. In many of the remaining examples, multiple mutations are involved in conferring resistance to the one antibiotic. The question arises whether epistasis between mutations or genes influences the relative fitness and evolutionary success of the resistant isolate (Fig. 5). Several studies of mutations conferring resistance to multiple antibiotics have shown epistasis affecting the relative fitness in different environments, with a tendency towards positive epistasis (Trindade *et al.*^2009^; Ward, Perron and Maclean 2009; Borrell *et al.*^2013^; Durao *et al.*^2015^). Epistasis is also significant for pairs of mutations that each confer resistance to the one antibiotic, and is a major determinant of the fitness cost of resistance mutations in different environments (Rozen *et al.*^2007^; Marcusson, Frimodt-Moller and Hughes 2009; Hall and MacLean 2011; Vogwill and MacLean 2015; Vogwill, Kojadinovic and MacLean 2016). In the case of resistance mutations affecting RNA polymerase (resistance to rifampicin) in *Pseudomonas*, the mechanism of epistasis was associated with individual mutations in *rpoB* having differential effects on global transcription patterns in different genetic backgrounds (Vogwill, Kojadinovic and MacLean 2016). There are also examples of epistasis involving combinations of chromosomal mutations and conjugative plasmids, including one of reciprocal sign epistasis, where a strain carrying both resistance determinants is fitter than strains carrying either of the single determinants (Silva *et al.*^2011^). Where the costs of plasmid carriage were measured, it was found that positive epistasis could minimise the cost associated with carrying multiple plasmids, and compensatory evolution, together with epistasis could increase the long-term stability of small, costly, non-conjugating plasmids (San Millan, Heilbron and MacLean 2014; San Millan *et al.*^2014^). Using an experimental evolution approach, it was also shown that genetic changes in both the host chromosome and on an antibiotic resistance plasmid contributed in combination to increasing plasmid persistence, with the magnitude of the combined changes versus the effects of the individual changes, supporting positive epistasis (Loftie-Eaton *et al.*^2016^).

Taken together, these various experiments show that in combination, resistance determinants, whether mutations or genes acquired by HGT, can often be associated with positive epistasis reducing the fitness costs of resistance, or increasing the level of resistance over that predicted by simple additivity (Huseby *et al.*^2017^). There is no reason in principle why similar mechanisms should not also operate to alter, and in many cases reduce fitness costs, or increase resistance level (Fig. 5), in clinical isolates.

### Heteroresistance causes phenotypic instability

**Figure 6.**
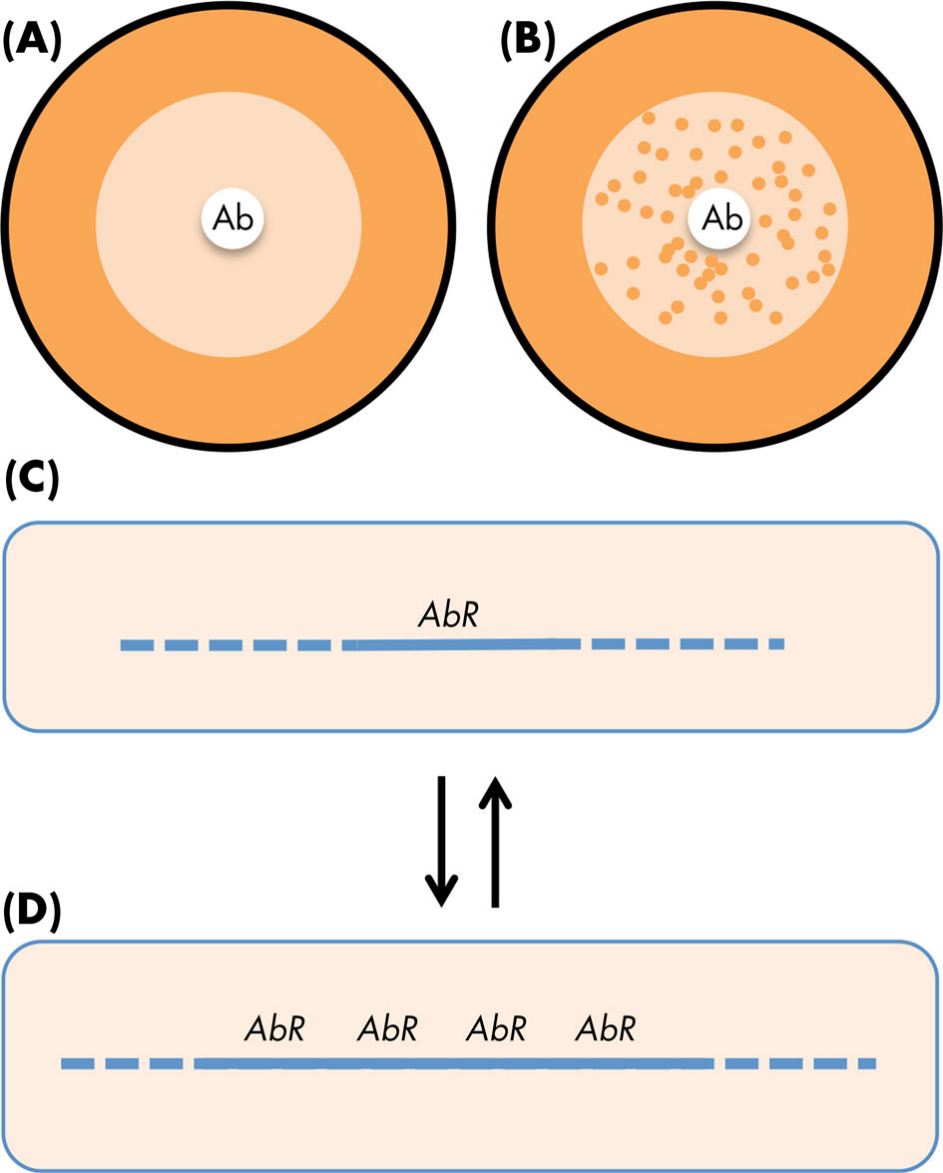
Heteroresistance causes phenotypic instability. Heteroresistance is a phenomenon of unstable (reversible) antibiotic resistance, reflecting the appearance of subpopulations of bacteria with different levels of susceptibilities in apparently isogenic populations. A disc diffusion assay with a zone of clearing (**A**). Heteroresistance in a disc diffusion assay (**B**), with colonies of resistant bacteria growing in the zone of clearing. A possible genetic explanation for heteroresistance (**C** and **D**). A gene responsible for the resistance phenotype (*AbR*) is subject to genetic amplification (a relatively frequent and reversible genetic phenomenon) that alters the level of drug susceptibility as a function of gene copy number.

Heteroresistance is a phenomenon where subpopulations of a seemingly isogenic bacterial isolate exhibit different susceptibilities to an antibiotic (El-Halfawy and Valvano 2015). The phenomenon was historically observed in both Gram-negative and Gram-positive species, associated with different antibiotics (Alexander and Leidy 1947; Sutherland and Rolinson 1964; Kayser, Benner and Hoeprich 1970) but the underlying mechanisms of resistance were unknown. A recent study of heteroresistance to colistin in *Salmonella* Typhimurium has identified a mechanism as unstable tandem amplifications of a chromosomal region that includes the gene, *prmD*, responsible for regulating proteins that modify lipid A (Fig. 6). In this case, the heteroresistance is explained by variable gene dosage of *prmD* in the different cell in the population, with subpopulations with different copy number of the gene showing different levels of colistin resistance (Hjort, Nicoloff and Andersson 2016). However, there are potentially many different mechanisms that could explain heteroresistance. Potential mechanisms (applicable to a strictly isogenic population of cells) include genetic (e.g. unstable genetic amplifications that affect susceptibility to the antibiotic or very high mutation rates), epigenetic (e.g. different levels of gene expression in different cells in a population) or non-genetic (e.g. if the local chemical environment differentially modulated the response of individual cells in the population to the antibiotic). In the clinical environment, any of these mechanisms, if it caused different cells to exhibit distinct antibiotic susceptibility phenotypes, could create a therapeutic problem because those subpopulations could potentially be selected to high frequency by exposure to inappropriate levels of an antibiotic. The potential to underestimate the problem caused by heteroresistance in the clinical microbiology laboratory is great. If only purified single clones from an infected patient are analysed for their spectrum of susceptibility, the danger is that heteroresistance will be significantly under-reported, whereas if all screening is made at the population level the costs and time taken to perform assays may be increased. The problem is not trivial and improving screening methods to detect subpopulations of resistant cells, in samples from, for example, *M. tuberculosis* patients where fluoroquinolone heteroresistance is a significant clinical problem (Mayer and Takiff 2014), or patients with heteroresistant vancomycin-intermediate *Staphylococcus aureus* (Gomes, Ward and LaPlante 2015), could have important consequences for determining the successful outcome of therapy for large numbers of infected patients worldwide.

### Globally successful resistant clones

**Figure 7.**
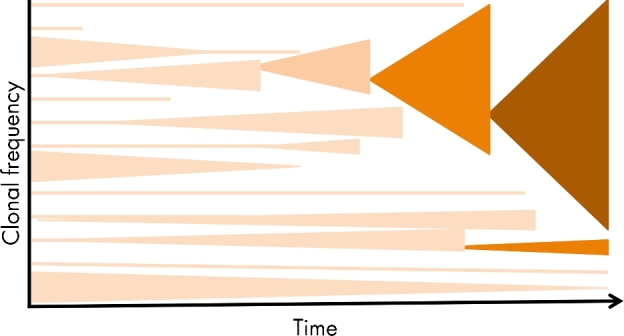
Globally successful resistant clones. Under selection the frequencies of some clonal lineages within a species will expand while others shrink and may go extinct. Clonal expansion may be favoured by mutation or HGT events improve transmission and resistance to antibiotics. Mechanisms and events associated with the global expansion and success of, for example, *E. coli* ST131 and *K. pneumoniae* ST258, are described in detail in the text.

Although resistance to an antibiotic can occur by mutation or HGT in any individual bacterium, what it observed for several important pathogens is that one or a few individual clones of the species appear to dominate globally. This suggests that the combination in one genome of a particular set of genes, including virulence factors and resistance determinants, may be major determinants of clonal expansion and global success (Fig. 7). Some successful genotypes probably pre-existed and achieved global prominence because of their ability, under selection, to acquire appropriate resistance determinants. In other cases, the successful genomes appear to have been created *de novo* by major genomic recombination events, again coupled with antibiotic selection and an ability to acquire the required resistance determinants. Here we discuss a few of these successful, MDR, clones. A caveat to this discussion is that publication bias may have led to some overestimation of the global success of these clones. In this respect, a recent meta-analysis of published data on the two most studied globally successful clones, *Escherichia coli* ST131 and *Klebsiella pneumoniae* ST258, concluded that there was insufficient data to conclude or reject their status as hyperendemic clones (Dautzenberg *et al.*^2016^).

#### Escherichia coli ST131

*Escherichia coli* lives as a commensal of the gastrointestinal tracts of humans and other animals but several variants have developed into significant pathogens through the gain and loss of genes (Croxen and Finlay 2010). One clonal lineage of *E. coli*, the sequence type 131 (ST131) from the phylogenetic group B2, has in the past decade become the predominant extraintestinal *E. coli* human pathogen globally, and is responsible for a range of infections, including community- and hospital-acquired UTIs and bacteraemia (Nicolas-Chanoine, Bertrand and Madec 2014). Almost all ST131 are resistant to multiple classses of antibiotics, including trimethoprim-sulfamethoxazole, amoxicillin and amoxicillin plus clavulanic acid, fluoroquinolones, and to extended-spectrum cephalosporins (Coque *et al.*^2008^; Nicolas-Chanoine *et al.*^2008^). What makes ST131 such a successful pathogen? Studies of ST131 from different geographical areas have revealed the presence of plasmids belonging to several different incompatibility groups (IncF, IncI, IncN, IncA/C, *pir-*type) that carry different β-lactamase genes, most frequently CTX-M-15 (but also other CTX-M-types, OXA-1, TEM-1 and NDM-1), as well as genes conferring resistance to a range of additional antibiotics (Naseer *et al.*^2009^; Woodford *et al.*^2009^; Partridge *et al.*^2011^; Bonnin *et al.*^2012^; Novais *et al.*^2012^; Nicolas-Chanoine, Bertrand and Madec 2014; Mathers, Peirano and Pitout 2015a). In this respect, ST131 differs from other B2 group isolates that have not acquired CTX-M-encoding plasmids. Extensive genome-wide analysis of historical isolates has provided insight into ST131’s origins and recent evolutionary history. This revealed a dominant variant carrying the *fimH30* allele (encoding the type I fimbrial adhesion genes) (Johnson *et al.*^2013^) and suggested that fluoroquinolone resistance evolved in ST131, in the early 2000s, in a single ancestor within the *H30* lineage referred to as *H30-*R (Price *et al.*^2013^). This same analysis also showed that 91% of the CTX-M-15 producing isolates of ST131 formed a single-ancestor subclone within the fluoroquinolone-resistance *H30-*R lineage, referred to as *H30*-Rx. These data supported the hypothesis that ST131 is a fluoroquinolone-resistant clone within which a subclone carrying CTX-M-15 has become globally prevalent by clonal expansion (Banerjee and Johnson 2014). It is not fully understood why ST131, alone among the B2 phylogenetic group of *E. coli*, has been so successful at acquiring and maintaining plasmids encoding CTX-M enzymes, although (Bonnin *et al.*^2012^) analysis of its evolutionary history strongly suggests that the emergence of the successful *H30*-Rx clade within ST131 was driven by the acquisition of a specific IncFII plasmid (Stoesser *et al*. 2016a). The success of ST131 is associated with a high level of transmissibility within household and hospital settings (Johnson *et al.*^2009^; Hilty *et al.*^2012^; Mathers, Peirano and Pitout 2015a), it has a prolonged carriage potential in long-term care patients compared with other ESBL *E. coli* strains (Overdevest *et al.*^2016^) and analysis of virulence gene profiles suggests that *H30* and *H30*-Rx subclones of ST131 are likely to be more virulent than non-*H30* ST131 isolates (Banerjee *et al.*^2013^). However, the identity of the actual virulence factors that make it so successful at colonising, persisting and transmitting from host to host remains a mystery (Nicolas-Chanoine, Bertrand and Madec 2014). Antibiotic treatment options for fluoroquinolone-resistant and ESBL-producing ST131 are very limited, and the resulting switch to the few remaining effective antibiotics increases the danger of selecting pan-resistant strains (Ochiai *et al.*^2008^). Carbapenems remain the preferred antibiotic of last resort but in recent years there have been reports of carbapenemase resistance in *E. coli* ST131 isolates from different countries around the world (Naas *et al.*^2011^; Accogli *et al.*^2014^; Cai *et al.*^2014^; O’Hara *et al.*^2014^; Peirano *et al.*^2014^; Johnson *et al.*^2015^; Ortega *et al.*^2016^; Stoesser *et al*. 2016b). Thus, in the 25 years, since the introduction of fluoroquinolones and extended-spectrum cephalosporins into clinical medicine, pan-resistant high-risk *E. coli* ST131 clones (Mathers, Peirano and Pitout 2015a; Stoesser *et al*. 2016a) have been selected and achieved a global spread. Detailed analysis of the genetic basis for clonally associated resistance in ST131 lineages may provide the knowledge base to devise intervention strategies (Stoesser *et al*. 2016a).

#### Klebsiella pneumoniae ST258

*Klebsiella pneumoniae* is a major cause of hospital-acquired pneumonias and bloodstream infections. Resistance to carbapenems, regarded as preferred last resort agents to treat *K. pneumoniae* (in contrast to colistin which has significant toxicity issues), has in recent years become a major global problem (WHO 2014). Resistance to carbapenems in *K. pneumoniae* is due to carriage of the plasmid-borne *Klebsiella pneumoniae* carbapenemases, KPC-2 and KPC-3 (Walther-Rasmussen and Hoiby 2007; Nordmann, Cuzon and Naas 2009), and the global spread of these genes is mainly associated with the pandemic strain ST258 (Mathers, Peirano and Pitout 2015b). The evolutionary history of ST258 is a facinating tale involving large chromosomal recombination events between different *K. pneumoniae* strains. Recombination between the chromosomes of *K. pneumoniae*, ST11 and ST442, together with the acquisition of an integrative conjugative element, ICEKp258.2, encoding a type IV pilus that facilitates attachment to surfaces and the exchange of plasmids, and a type III restriction-modification system that could restrict the types of plasmids that could be exchanged, resulted in the creation of ST258 clade II (Chen *et al*. 2014a). Subsequently, ST258 (clade I) evolved from clade II by an additional chromosomal recombination event, replacing the *cps* region of the chromosome (capsular polysaccharide) with an equivalent region from another *K. pneumoniae* strain, ST42 (Chen *et al*. 2014a). The success of ST258 is undoubtedly multifactorial but one critical feature seems to be its strong association with MDR plasmids (Deleo *et al.*^2014^). ST258 (clades I and II, both of which contain ICEKp258.2) is specifically associated with KPC and narrow-host-range IncF plasmids (Chen *et al*. 2014b), whereas its immediate ancestor ST11 (which lacks ICEKp258.2) is associated with various carbapenemases carried on broad-host-range plasmids (Voulgari *et al.*^2014^; Liu *et al.*^2015^). Sequence analysis of ST258 clones has revealed four different plasmids and 24 different resistance genes covering all major classes of antibiotic (Villa *et al.*^2013^, ^2014^; Lee *et al.*^2014^), and reduced expression of porins, leading to colistin resistance (Clancy *et al.*^2013^).

#### Salmonella enterica serovar Typhimurium DT104

*Salmonella enterica* is one of the most common food-borne pathogens, and the MDR DT104 clone is a major public health concern. It carries genes for resistance to ampicillin, chloramphenicol, streptomycin, sulfonamides and tetracycline. MDR in DT104 is the result of the acquisition by HGT of a 13-kb gene cluster containing, in addition to resistance genes, a class I integron. This MDR region recombined into a region on the chromosome of DT104 known as the *Salmonella* genomic island I (SGI1) (Leekitcharoenphon *et al.*^2016^). SGI1 itself is an integrative mobilisable element carrying several virulence factor genes (Doublet *et al.*^2005^). DT104 originated from a drug-susceptible phage type that probably had a unique genetic origin as a clone within the *Salmonella* Typhimurium sequence type 19 (Matiasovicova *et al.*^2007^; Cooke *et al.*^2008^) where it acquired several virulence factors that may have contributed to its endemic abilities after acquisition of multidrug-resistance (Cooke *et al.*^2008^).

There are significant differences and some similarities between the successful high-risk *E. coli* ST131, *Salmonella* DT104 and the *K. pneumoniae* ST258 clones. ST131 seems to have originated from a pre-existing drug-susceptible clone that initially evolved, by the occurrence of multiple chromosomal mutations, under selection pressure to resist fluoroquinolones, and subsequently became a fully fledged high-risk clone, by acquiring IncF plasmids encoding CTX-M β-lactamases, under the selection pressure to resist cephalosporins (Stoesser *et al.*2016a). DT104 also seems to have emerged as a threat only after is acquired by HGT the MDR gene cluster into SGI1 (Leekitcharoenphon *et al.*^2016^). It has been speculated that the relative success of ST131, compared to other ExPec *E. coli* strains, may have been largely due to inherent properties of its genomic makeup: the presence of a specific set of virulence genes, a set of genes conferring a large metabolic potential, and genes conferring an enhanced ability to make biofilms, together with the possibility that the evolution of fluoroquinolone resistance mutations somehow reduced the carriage cost of IncF CTX-M-producing plasmids (Mathers, Peirano and Pitout 2015b). In this sense, ST131 and DT104 may have been an accidents waiting to happen, and their emergence as global MDR clones was driven directly by antibiotic selection. ST258 in contrast seems to have emerged as a high-risk clone only after a series of major chromosomal recombination events, creating a hybrid between ST11 and ST442, together with recombination into the chromosome of ICEKp258.2 (Chen *et al*. 2014a). These recombination events created a hybrid strain receptive to IncF plasmids that express KPC enzymes, plasmids that also carry genes conferring resistance to many other antibiotics (Chen *et al*. 2014b). We do not yet know how frequently such chromosomal hybrids occur naturally in *Klebsiella*, so we can only speculate as to whether it was exceptional bad luck to select a clone with the properties of ST258, or whether this also was a high-probability accident-waiting-to-happen, and selected to a high frequency by antibiotic usage.

#### Pseudomonas aeruginosa

The previous examples concern globally distributed pathogens with a high level of access to humans and that are easily transmitted in both hospital and community settings. *Pseudomonas aeruginosa*, in contrast, is a soil bacterium that is nevertheless a significant opportunistic pathogen of the lungs of humans with the genetic condition cystic fibrosis (CF) (Winstanley, O’Brien and Brockhurst 2016), and a major cause of nosocomial infections, including ventilator-associated pneumonia and burn wound infections (Vincent 2003). CF patients are initially infected with whatever strain of *P. aeruginosa* is carried by someone in their close environment, and the infecting strain often persists and adapts by accumulation of mutations to establish a long-term drug-resistant infection in the patient (Cramer, Wiehlmann and Tummler 2010). The low population density of CF patients, and the introduction of measures to reduce transmission at centres where patients are treated, contributes to the discrete, patient-specific, genetics of most CF infections. However, even within an environment not conducive to the development of a globally spread high-risk clone, there is evidence that at least some specific highly transmissible clones are emerging. A clone designated as Liverpool epidemic strain (ST146) is highly transmissible and appears most likely to infect patients already infected with another strain, suggesting it has a high competitive ability and high transmissibility (McCallum *et al.*^2002^; Fothergill, Walshaw and Winstanley 2012). Among the noscomial infections caused by *P. aeruginosa* in non-CF patients, there is increasing evidence that a few high-risk MDR clones are dominating and spreading globally (Oliver *et al.*^2015^). Among these high-risk MDR/XDR clones, ST235 and ST111 are each distributed globally and each have acquired multiple different β-lactamases (Oliver *et al.*^2015^).

The high frequency and global prevalence of specific problematic clones such as ST131, DT104 and ST258, and the various high-risk *P. aeruginosa* clones might motivate developing targeted therapeutic solutions, for example, vaccine production, or drugs targeting of novel factors associated with these high-risk clones.

#### Streptococcus pyogenes

The group A streptococci (GAS) are human pathogens that colonise the throat or skin. GAS can cause invasive infections, including septic shock, necrotising fasciitis and streptococcal toxic shock syndrome, with an average associated mortality of 25% (Cole *et al.*^2011^). There has been a global increase in invasive GAS infections since the 1980s, associated with the emergence of a clonal group of strains with the M1T1 serotype (Aziz and Kotb 2008; Cole *et al.*^2011^). Genomic sequencing of several thousand strains of serotype M1 has revealed that the epidemic virulent clone evolved from a single bacterial cell in a stepwise manner, by a combination of mutation and HGT, involving multiple acquisitions of DNA encoding virulence factors, including secreted toxins that greatly increase the severity of the infection (Nasser *et al.*^2014^). Antibiotic resistance in the classical sense (resistance mutations or resistance genes affecting clinical antibiotics) is not associated with GAS, and antibiotic therapy is generally effective against non-invasive infections. However, invasive GAS infections are often so aggressive that antibiotic therapy may be insufficient or applied too late to be effective. One possible contributing factor to this failure of antibiotic therapy in the case of the M1T1 clone is that in addition to increasing virulence, the M1 protein also mediates resistance to the human cathelicidin antimicrobial peptide LL-37 (LaRock *et al.*^2015^), a part of the human innate antimicrobial defence system (Andersson, Hughes and Kubicek-Sutherland 2016).

#### Streptococcus pneumoniae

Clonal diversification and subsequent expansion of successful variants are the cause of major problems in tackling invasive serotypes *S. pneumoniae*. Diversification in *S. pneumoniae* is associated with a high frequency of recombination with DNA from genetically related bacteria (Spratt, Hanage and Feil 2001). Pneumococci inhabit the nasopharynx where transformation-competent cells can kill non-competent cells, causing the release DNA that increases the rate of HGT between different strains of *S. pneumoniae*, and related commensal species (Johnsborg *et al.*^2008^). *Streptococcus pneumoniae* are classifiied into at least 90 different serotypes based on differences in it capsular polysaccharide, an important virulence determinant (Geno *et al.*^2015^). Some specific serotypes are associated with a high propensity to cause invasive disease, whereas other serotypes are associated with healthy carriers (Brueggemann *et al.*^2004^; Henriques-Normark *et al.*^2008^). Penicillin is the antibiotic of choice to treat pneumococcal infections but penicillin non-susceptible pneumococcal (PNSP) clones have evolved, largely through multiple HGT recombination events. MDR pneumococci are strongly associated with only a few of the many serotypes, and the relative frequencies of these serotypes differ geographically, and temporally as a function of when the isolates have been sampled (Kim *et al.*^2016^). The high burden caused by invasive pneumococcal infections, particularly in children and the elderly (meningitis and bloodstream infections) motivated the introduction of widespread vaccination, targeting the capsular polysaccharide of the most frequent virulent serotypes. The seven-valent pneumococcal conjugate polysaccharide vaccine (PCV-7), for use in infants and young children, targeted seven prevalent serotypes and was introduced in 2000. It initially caused a significant reduction in the incidence of invasive disease caused by the targeted serotypes (Whitney *et al.*^2003^) but its use was soon associated with an increase in the frequency of a non-vaccine serotypes (Hicks *et al.*^2007^). Vaccine-escape strains evolved by an HGT event that simultaneously changed the serotype and conferred a PNSP phenotype (Brueggemann *et al.*^2007^), or by clonal expansion of previously minor lineages (Henriques-Normark *et al.*^2008^). The subsequent introduction of vaccines targeting 10 or 13 different serotypes, PCV-10 and PCV-13, helped to further reduce mortality but were also been associated with shifts in the population structure to non-vaccine serotypes (Pittet and Posfay-Barbe 2012), although there has been a significant overall decrease in invasive infections and in antimicrobial-resistant pneumococci (Torres *et al.*^2015^; Kim *et al.*^2016^). The huge intrinsic variation in the pneumococcal population, the high frequency of HGT and the potential for rapid changes in the global frequency of different clonal types suggest that surveillance to monitor and re-evaluate control measures will be essential (Cohen, Biscardi and Levy 2016).

## CONCLUSIONS AND PERSPECTIVES

A central objective of genetics is to understand how a phenotype is generated from the interplay between a genotype and a set of environmental conditions, and to develop methods that allow us to predict phenotypes from genetics and DNA sequences alone. With regard to antibiotic resistance, and the emergence and spread of successful clones, recent findings show clearly that this objective is complicated by the fact that the phenotypic expression of a given resistance gene/mutation can be modified and changed because of alterations in environmental conditions and the genetic context. Continuing advances in genomic sequencing technology, and in the fields of bioinformatics, experimental evolution and genetics, will continue to provide increased power to link genotypes with phenotypes. The importance of acquiring and being able to apply this knowledge cannot be underestimated. As we face into a future where resistance to many currently used antibiotics increases to a point where they risk becoming useless, infection control will increasingly depend on the development of novel classes of antibiotics for which there is no pre-existing resistance in clinical pathogens. One of the major challenges will be to successfully predict resistance by HGT, and resistance evolvability, early in drug development, so that effective measures can be implemented (e.g. to focus development resources on those drug candidates less prone to resistance development by HGT, and to prepare adequate antibiotic stewardship programmes prior to marketing a novel drug class). A second area where a deeper understanding of genotype–phenotype relationships will be valuable is in designing improved regimens for antibiotic therapy that can exploit weaknesses in bacterial resistance phenotypes, and reduce the use of inappropriate therapies that might select resistance. To address this complexity, future work needs to more systemically generate genotype-phenotype maps that take into account the variable in vivo conditions in which bacterial pathogens reside, the genetic variability of natural strains, the potential for HGT from the pan-genome, and the evolvability of foreign ‘resistance’ genes after transfer into a novel genetic environment.

***Conflict of interest.*** None declared.
